# An altered endometrial CD8 tissue resident memory T cell population in recurrent miscarriage

**DOI:** 10.1038/srep41335

**Published:** 2017-01-23

**Authors:** J. H. Southcombe, G. Mounce, K. McGee, A. Elghajiji, J. Brosens, S. Quenby, T. Child, I. Granne

**Affiliations:** 1 Nuffield Department of Obstetrics and Gynaecology, University of Oxford, Level 3 Women’s Centre, JR Hospital, Headley Way, Headington, Oxford, OX3 9DU, UK; 2 Nuffield Department of Obstetrics and Gynaecology, Oxford Fertility and Institute of Reproductive Sciences, University of Oxford, Level 3 Women’s Centre, JR Hospital, Headley Way, Headington, Oxford, OX4 2HW, UK; 3 Division of Biomedical Sciences, Clinical Science Research Laboratories, Warwick Medical School, University of Warwick, Coventry CV2 2DX, UK; 4 Tommy’s National Miscarriage Research Centre, University Hospitals Coventry & Warwickshire, Coventry CV2 2DX, UK

## Abstract

When trying to conceive 1% of couples have recurrent miscarriages, defined as three or more consecutive pregnancy losses. This is not accounted for by the known incidence of chromosomal aneuploidy in miscarriage, and it has been suggested that there is an immunological aetiology. The endometrial mucosa is populated by a variety of immune cells which in addition to providing host pathogen immunity must facilitate pregnancy. Here we characterise the endometrial CD8-T cell population during the embryonic window of implantation and find that the majority of cells are tissue resident memory T cells with high levels of CD69 and CD103 expression, proteins that prevent cells egress. We demonstrate that unexplained recurrent miscarriage is associated with significantly decreased expression of the T-cell co-receptor CD8 and tissue residency marker CD69. These cells differ from those found in control women, with less expression of CD127 indicating a lack of homeostatic cell control through IL-7 signalling. Nevertheless this population is resident in the endometrium of women who have RM, more than three months after the last miscarriage, indicating that the memory CD8-T cell population is altered in RM patients. This is the first evidence of a differing pre-pregnancy phenotype in endometrial immune cells in RM.

The mucosal surface of the uterus, the endometrium, is populated by innate and adaptive immune cells that provide a first line of defence towards pathogens. However, its primary function is to facilitate embryo implantation, when immune cells must tolerate the semi-allogeneic fetus without a significant loss of host immunity^1^. Similar to other mucosal tissues, most endometrial CD8-T cells have an effector memory phenotype^2^,^3^. Recent murine studies have led to a new understanding of memory CD8-T cells in tissues. The majority of these cells have been characterized as ‘Tissue Resident Memory’ (Trm) cells, which provide the typical rapid effector responses associated with memory cells^4^, but crucially they do not recirculate. They are therefore independent of lymphoid and peripheral blood memory T cell populations^5^. CD8-Trm remain in tissues despite the lack of persistent antigen^6^, an attribute associated with the expression of CD69 and CD103^7^,^8^. CD69, the classical early activation marker, also has a reciprocal relationship with sphingosine-1-phosphate receptor-1 (S1PR1)^9^; CD69 upregulation leads to S1PR1 downregulation, which prevents cell egress from both lymphoid and non-lymphoid organs following sphingosine-1-phosphate (S1P) gradients^10^,^11^. CD103 is the ligand for e-cadherin^12^, which is highly expressed on epithelial cells in mucosal tissues^13^.

In humans, few studies have analysed tissue memory CD8-T cell responses, due to the limitation of acquiring normal human tissues for research purposes, although detailed analyses of CD8-Trm subsets have been performed on mucosal tissues (lung, jejunum, ileum, colon) from cadaveric organ donors^3^,^14^,^15^. Similar to murine studies, CD69 and CD103 are highly expressed by mucosal human memory CD8-T cells, in contrast to circulating memory CD8-T cells^3^,^14^,^15^. While studies have investigated memory T cells in the decidua (the modified endometrium during pregnancy) using tissues obtained from elective abortions or term pregnancies^16^,^17^, a detailed analysis of residency markers on CD8-T memory cells in non-pregnant endometrium, has not yet been performed. Here we isolate and phenotype endometrial CD8-T cells in the mid-luteal phase of the menstrual cycle (the time relevant for embryo implantation) and demonstrate that these cells are altered in women who have experienced recurrent miscarriage (RM), a condition hypothesised to have an immune mediated mechanism.

Although the commonest cause of sporadic miscarriage is fetal aneuploidy, RM, 3 or more consecutive miscarriages, often occurs with euploidic pregnancies^18^. RM affects 1% of couples, an incidence three times higher than expected by chance alone, further suggesting RM is a distinct clinical entity^18^. RM associated may be attributed to, or associated with a number of underlying factors including uterine structural abnormalities, autoimmune conditions (for example thyroid autoimmunity) and inherited or acquired thrombophilias. Despite investigation however, the majority of RM remains unexplained.

In the first days of pregnancy, the embryo attaches to the endometrial epithelial wall and then implants into the endometrial stroma; as early pregnancy continues, trophoblast cells invade through the endometrial stromal layer establishing placentation. The trophoblast cells that invade the endometrium, specifically extravillous cytotrophoblast, come into direct contact with T cells, but they are inhibited by factors secreted from both the trophoblast and decidualised endometrium, such as indoleamine 2,3-dioxygenase and soluble HLA-G, implying their functions can be dangerous to the developing conceptus^19^. Despite the release of these factors, inhibition is not complete and T cell responses directed towards the conceptus are formed; peripheral and decidual T cells restricted by male-specific minor histocompatibility (HY) antigens can be detected throughout pregnancy and postpartum^20^,^21^,^22^ and T cells may be activated by other factors such as NK cell receptor ligands^23^. There is evidence that an immunological memory response to pregnancy can be generated as secondary RM is more frequent after a first successful male, rather than female pregnancy^24^; and pregnancies ending in miscarriage rather than live birth are associated with an increased risk of further miscarriage^25^. Therefore we hypothesised that endometrial CD8-T cells are Trm whose phenotype and function may be altered in unexplained RM.

## Results

### Endometrial immune cell proportions unaltered in RM

**Figure 1:**
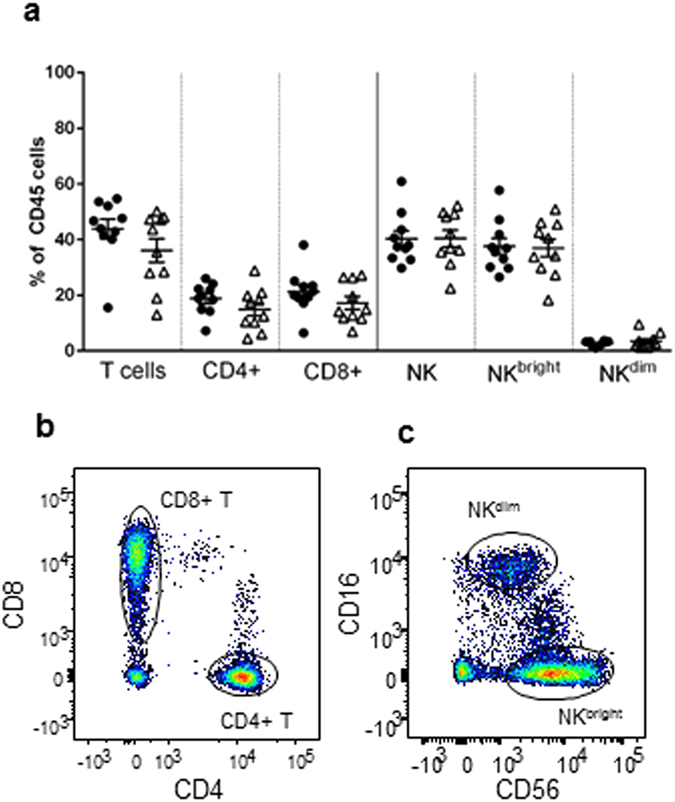
Phenotypic analysis of isolated CD45+ cells in the endometrium by flow cytometry. Cells were isolated from the endometrium and stained with CD45-AlexaFluor-647, CD3-PECy5, CD4-FITC, CD8-PE, CD56-PECy7, CD16-APCCy7 antibodies. Endometrial immune cells from 10 control women (•) or 10 women with RM (Δ) were characterised and proportions of each cell population in the CD45+ lymphocyte gate displayed (**a**). Data displayed as mean ± S.E.M. Representative flow cytometry staining of endometrial CD4 and CD8 T cell populations in the CD45+/CD3+ cells (**b**) and NK^bright^ (CD56^bright^/CD16−) and CD56^dim^ (CD56+/CD16+) in CD45+/CD3− gating (**c**).

Endometrial biopsies were obtained from RM patients and controls during the ‘window of embryo implantation’, 7–11 days after the luteinising hormone surge. Tissues were digested and cells analysed by flow cytometry. The majority of CD45+ cells were T and NK cells (Fig. 1a), with similar percentages of T cells (mean 43.85%) and NK cells (40.27%) in controls. Similar proportions of CD4+ and CD8+ cells were detected (18.77% and 21.31% of the total CD45+ cells, representative example Fig. 1b) and the majority of NK cells were an NK^bright^ (CD3−/CD56^bright^/CD16−) phenotype (93.3%) rather than NK^dim^ (CD3−/CD56+/CD16+) (representative example Fig. 1c). This is in contrast to peripheral blood where CD4+ cells comprise two thirds of the T cell population and NK cells are less than 10% of the leukocyte population that display an NK^dim^ phenotype (Supplementary Figure 1a). Our analysis of endometrial biopsies therefore have minimal blood contamination and are of cells that reside within the tissue. Of note, no differences in the proportions of T cells or NK cells within the endometrium were seen between RM and controls (Fig. 1).

### A modified endometrial CD8-Trm phenotype in RM

**Figure 2:**
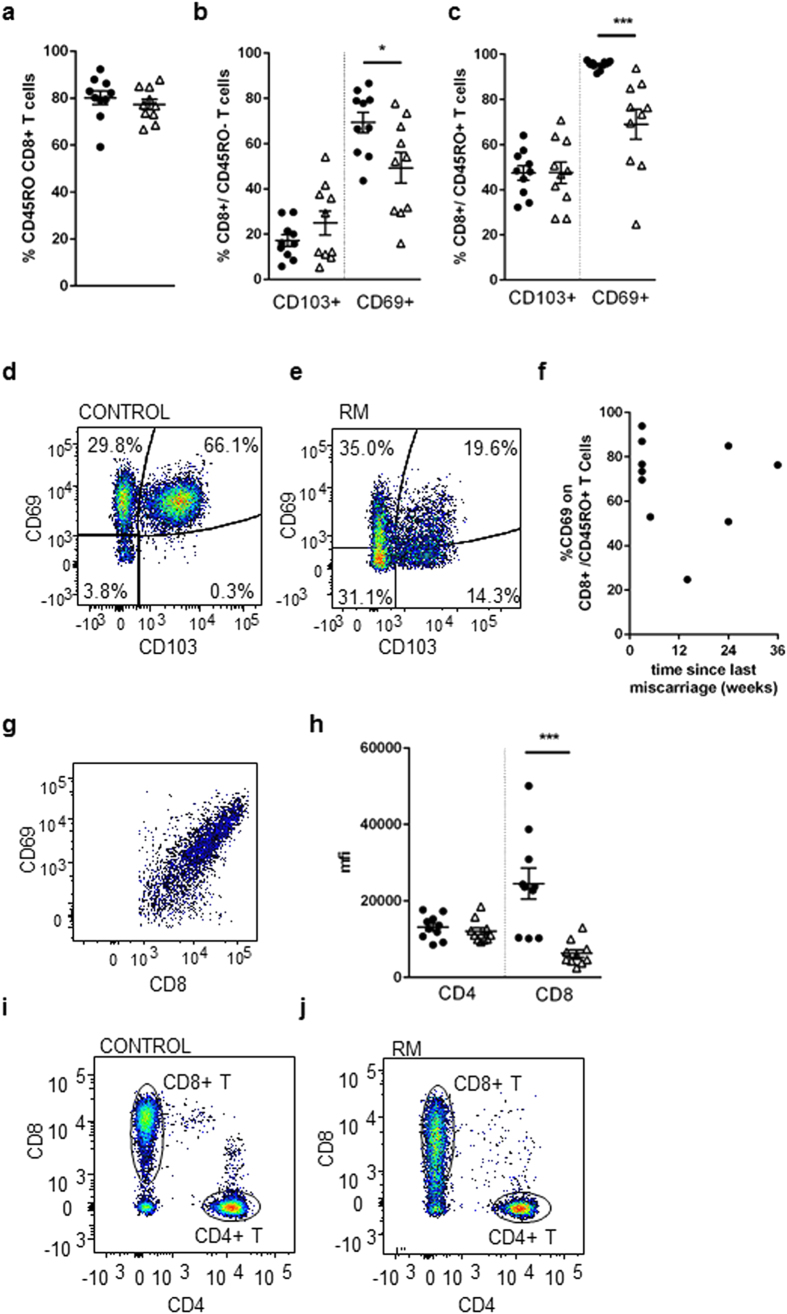
Phenotypic analysis of CD8+ endometrial T cells by flow cytometry. Endometrial CD3+CD8+ T cells from 10 controls (•) or 10 RM (Δ) were stained with antibodies CD3-PeCy5, CD8-PECy7, CD45RO-APCCy7, CD103-FITC, CD69-PE with FMO controls. The proportion of cells expressing CD45RO (**a**) and CD103 and CD69 expression was determined on CD45R0−’ve (**b**) and CD45RO+ ’ve (**c**) CD3+/CD8+ T cell populations. Representative example of flow cytometry staining profile of CD103 and CD69 on control (**d**) and RM (**e**) CD8+CD45RO+ cells. CD69 expression on CD8+CD45RO+ cells in RM women versus time since last miscarriage (weeks) (**f**) and showing CD8 and CD69 correlative expression (**g**). The mean fluorescence intensity of the CD4 and CD8 co-receptor on samples was also calculated (**h**), representative flow cytometry images of CD8 and CD4 staining on CD3-T cells from control (**i**) and RM (**j**) women. Bars represent mean+/− S.E.M. For significance values a Student’s *t*-test comparison of controls and RM was performed.

Analysis of CD3+/CD8+ cells for markers of memory and tissue residency was performed. The majority of cells in the endometrium were CD45RO+’ve: controls (80.2 ± 2.9%) and RM (77.3 ± 2.2%) (Fig. 2a). This is in contrast to blood where approximately 20% of the circulating CD3+/CD8+ T cells express CD45RO (Supplementary Figure 1b). CD69 and CD103 expression on non-memory CD45RO−‘ve (Fig. 2b) and memory CD45RO+’ve (Fig. 2c) endometrial CD3+/CD8+ T cells were assessed. In controls the majority of non-memory cells expressed CD69 but not CD103. CD103 expression was higher on the total CD8-T cell CD45RO+ (47.40 ± 3.2%) versus CD45RO− (17.16 ± 2.6%) populations (p < 0.0001); CD69 was higher on the total CD45RO+ (95.3 ± 0.6%) versus CD45RO− (69.4 ± 4.5%) population, indicating memory CD8-T cells are more likely to be retained in the tissue than non-primed cells. For comparison, peripheral blood CD3+/CD8+ cells from non-pregnant donors were also analysed: CD103 expression was 0.7 ± 0.2% CD45RO− and 6.0 ± 1.7% on CD45RO+ cells and CD69 expression was 7.9 ± 3.1% on CD45RO− and 16.4 ± 4.3% on CD45RO+ (Supplementary Figure 1c,d). A differing profile of tissue resident marker CD69, but not CD103, was seen in both non-memory and memory CD8-T cell populations in RM. A lower proportion of CD45RO− cells were CD69+ (p < 0.05) (Fig. 2b). RM patients also had fewer CD69+CD45RO+/CD8-T cells (p < 0.001) (Fig. 2c), representative flow cytometry shown in Fig. 2d versus 2e. Decreased CD69 was not an acute response to miscarriage as there was no correlation between CD69 levels and time since the last miscarriage (Fig. 2f). Intriguingly, low CD8 levels were correlated with lower CD69 levels–representative example is shown in Fig. 2g. Significantly lower levels of CD8 expression were also detected on RM T cells compared to controls, whereas CD4 levels were similar between groups (Fig. 2h), representative flow cytometry images from controls (i) and (j) shown.

### Endometrial CD8-Trm have increased activation potential, but controls and RM have similar capacity to produce IFNγ

**Figure 3:**
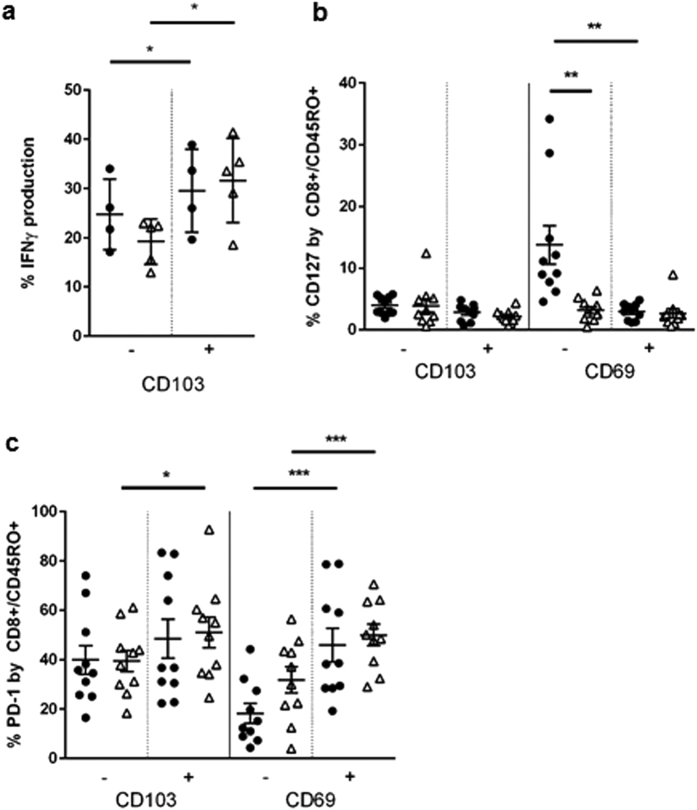
Activation potential of endometrial Trm in controls and RM. Endometrial biopsy digests were stimulated with CD3/CD28 beads for 5 hours, with the addition of brefeldin A after 1 hour, cell surface markers CD45/CD3/CD69/CD103 were stained then intracellular IFNγ expression was determined on control (•) and RM samples (Δ) (**a**). CD127 (**b**) and PD-1 (**c**) expression levels on CD103+/− and CD69+/−CD8-T was analysed by staining with CD3-PeCy5, CD8-PECy7, CD45RO-APCCy7, CD103-FITC, CD69-PE and CD127-AlexaFluor647 or PD-1-AlexaFluor647 with FMO controls. Bars = mean +/−S.E.M.

CD8-Trm are known to rapidly produce IFNγ upon stimulation^4^, and we hypothesised that the low expression levels of CD8 and CD69 would result in a lower capacity to produce IFNγ as the change infers a regulatory phenotype^26^,^27^. We stimulated endometrial T cells with CD3/CD28-beads and analysed their potential to produce IFNγ by intracellular staining and flow cytometry (Fig. 3a). Background levels of CD8-T cells IFNγ production were less than 1% and similar between groups (data not shown). We found that endometrial CD8 cells responded to stimulation with 10–40% of CD8-T cells producing IFNγ within five hours of stimulation. A greater proportion of CD103+ cells produced IFNγ in both controls and RM. We could not analyse if CD69− versus CD69+ cells, nor CD8 low versus high cells, had altered potential because both markers were upregulated by our isolation procedure and during the five-hour co-culture, even in the absence of TCR stimulation (data not shown). However, the total CD8-Trm cell population from controls and RM patient expressed similar amounts of IFNγ, therefore the phenotypic change seen in RM does not effect this particular function. To further probe the nature of the CD69− versus CD69+ populations two markers of activation status were measured: CD127 and PD-1 (Fig. 3b,c). CD127 is the IL-7 receptor, a cytokine required for homeostasis that is down regulated on IL-7 stimulated cells^28^ and on activated effector cells^29^. PD-1 is an inhibitory molecule expressed by T cells and is the receptor for PD-L1^30^. CD127 expression was generally low (Fig. 3b), but cells lacking CD69 had higher CD127 expression than CD69+ cells (p < 0.01); this increase was not noted in the CD69− population in RM that expressed significantly lower CD127 (p < 0.01). PD-1 expression was highly variable between patients (Fig. 3c), in controls and RM PD-1 was lower on CD69− cells than cells expressing CD69 (P < 0.001). No differences were seen between controls and RM. PD-1 expression also was higher in CD103+ versus CD103− cells in RM (P < 0.05).

## Discussion

Similar to other human mucosal tissues^3^, >90% of CD8-T cells in the endometrium are CD45RO positive. This observation is in agreement with an earlier study that found few endometrial CD45RA positive CD8-T cells–the majority were CD45RA−/CCR7− indicative of the effector memory subset^2^. We hypothesised that this predominant CD8-T memory cell population would be Trm and therefore analysed expression of two key proteins that are directly involved with immune cell retention in tissues, CD103 and CD69. We found that the expression of both proteins was higher in the CD45RO+ versus CD45RO− population: CD103 (50% versus 20%) and CD69 (>90% versus 60%). Endometrial CD8-T cells therefore use both mechanisms of tissue retention and memory cells have a greater retention capacity. The ligand for CD103, E-cadherin, is highly expressed on endometrial glands and surface epithelium^31^, thus providing a mechanism for CD103+ CD8-T tissue residency, as is the case for the intestine^12^. The majority of memory CD8-T cells expressed CD69, which is similar to the gut^3^. Endometrial CD8-T cells were previously reported to express CD69 in the proliferative and luteal phase of the menstrual cycle^32^. Similarly, CD8-T cells in the first trimester decidua (a modified endometrium which produces factors such as hormones and cytokines to support pregnancy) have expression of CD69 similar to our findings here^33^. Although high CD69 expression can indicate cellular activation^34^, it now seems clear that CD69 expression also marks resident endometrial CD8-T cells that remain in the tissue in early pregnancy.

The proportions of endometrial T cells (both CD4-T and CD8-T) were similar in RM and controls, which is in agreement with a previous immuno-histological study^35^. In addition we analysed NK cells, NK^bright^ cells which have a classical CD56^bright^/CD16^negative^ expression are the most abundant population of immune cells in the decidua in early pregnancy and produce cytokines to assist placentation^36^. Here we find similar proportions of T cells and NK cells in the non-pregnant endometrium. NK cells are often implicated in the pathology of RM as increased numbers are reported in the RM endometrium^35^, however we found similar numbers of NK between the two groups. This is probably because we determine the percentage of NK^brights^ within the lymphocyte population (CD45+) whereas the reported increase in NK cell numbers are found by immunohistochemical studies that report CD56 expressing cells as a proportion of total stromal cells.

Although sporadic miscarriage may be associated with acute infection, epidemiological data do not suggest a link between infection and RM^37^ and in fact all endometrial biopsies used in this study were taken at least 3 months from the last miscarriage. Consistent with this, RM patients did not have differing proportions of CD45RO− CD8-T cells. CD103 expression was similar between groups, however CD69 expression was significantly reduced in RM, which correlated with decreased expression of the TCR-MHC class I co-receptor CD8. As both the ratio of CD4:CD8 T cells and the proportions of memory CD8-T cells are similar, it is likely that they are still being retained within the tissue, rather than being an additional infiltrating population. CD103 expression may compensate for the loss of some of the CD69 preventing cell egress, indeed we identified a population of CD103+/CD69− cells in RM endometrium that is not present in control tissues (see representative flow cytometry images in Fig. 2d versus 2e). It is likely that another mechanism of tissue retention for the CD103−/CD69− cells exists, and we find that CD8-T cell CD69 levels can still be reduced up to 3 years since the last miscarriage in RM, therefore the lack of this marker does not necessarily lead to egress.

Reduced CD8 expression is observed transiently on acutely activated effector cells^38^, however CD3 expression on the RM CD8-T cells is the same as controls (data not shown), indicating cells are not activated. Reduced CD8 expression is also found after repeated antigen exposure and is associated with decreased T cell responsiveness and a regulatory phenotype that may confer increased peripheral tolerance^26^,^39^. However, this evidence is derived from murine studies and focuses on peripheral blood or lymphoid compartments; whether reduced CD8 expression has a functional relevancy to endometrial memory CD8-T cells merits further investigation. Additionally, reduced CD8 co-receptor expression was associated with reduced CD69 expression. CD69 is a type II c-type lectin, similar to NK receptors such as NKG2D and CD94^40^. CD69 was originally described as a marker of T cell activation, however other functions have since been ascribed. For example, CD69 is also regulatory and mice lacking CD69 show heightened autoimmunity and clear tumors more efficiently^27^. Reduced expression of both CD8 and CD69 in RM could infer a functional pathological mechanism. We stimulated endometrial CD8-Trm cells through TCR engagement (CD3/CD28 beads for 5 hours), and assessed IFNγ production by intracellular flow cytometry, 10–40% of CD8 cells produced IFNγ. CD8-Trm in the non-pregnant murine female reproductive tract secrete IFNγ when infected with vaginal vaccinia infection^41^, which triggers a tissue wide anti-viral state whereby B, dendritic and NK cells are recruited and activated and human endometrial CD8-Trm may have similar bystander communication. In our assay we found that RM patient CD69 and CD8 expression levels returned to the same high expression as on as control cells, even in the absence of cellular stimulation, presumably due to stresses caused by the isolation procedure and duration of culture. This made it difficult to study the function of the RM CD69−/CD8low cell phenotype. We could however compare the differences between CD103− and CD103+ populations as this marker did not change. CD8 cells expressing CD103 produced higher levels of IFNγ, similar to seen in the skin^42^.

We were able to study if lower CD69 expression is associated with reduced functional potential with reference to CD127 expression. CD127 expression was low in endometrial CD8-T cells, in contrast to the jejunum, ileum or colon mucosa^14^. Low levels of CD127 are also found in CD8-Trm in the lung^43^, which may be associated with decreased maintenance of memory cells in this tissue. Further work is required to determine the survival capacity of human endometrial CD8-Trm as other signals could be responsible for their proliferation and survival like those postulated for lung Trm^44^. CD127 was significantly higher on CD69− versus CD69+ cells (p < 0.01) in controls, indicating CD69+ Trm are less likely to respond to IL-7 than other memory cells, which could indicate they are less likely to survive.

The finding of lower PD-1 on these CD69− cells indicates they are more resistant to inhibition by its ligand PD-L1. The CD69− population identified in control endometrium is however a minority subset, albeit one that has greater activation potential. In RM this population is significantly more predominant, and these cells also have lower CD127 expression. Therefore the CD69− memory cells present in RM may lack the potential for long term survival. Samples used in this study were all taken at least 3 months after the last miscarriage, therefore the altered phenotype seen in RM must have survival and retention signals not afforded by IL-7 signalling and CD69 tissue retention if they either pre-exist the last miscarriage or are triggered by the miscarriage itself. Alternatively they may be a population expanded by an unknown stimuli post miscarriage.

No other study directly compares pre-pregnancy endometrial CD8-T cell responses between normal and RM women; all previous studies have been restricted to decidual CD8-T cell clones generated from tissues obtained during miscarriage surgery. In those experiments the clones have reduced capacity to generate IL-4 which may be detrimental to pregnancy success^45^, but it is not clear if this is a response to miscarriage rather than a cause of miscarriage, nor if this T cell phenotype is long-lived. Here we describe the first evidence of an altered peri-implantation CD8-T cell phenotype in RM. Future studies are warranted to identify the trigger for the altered CD8-Trm phenotype associated with RM and if this reflects an altered cell repertoire with antigen specificity, and to identify if this has further functional relevancy to future pregnancy outcomes.

## Methods

### Ethical Approval

The study was approved by the Oxford Research Ethics Committee C (ref:08/H0606/94). Methods were carried out in accordance to guidelines and regulations. All participants gave written informed consent.

### Patient Characteristics

Endometrial samples were obtained from ovulatory cycles and recruits were at least three months post miscarriage or hormonal treatment. Samples were taken between 7–11 days post LH pre-ovulatory surge; normal endometrial morphology and timing to the secretory phase was confirmed by H + E staining (data not shown). All women with RM had normal thyroid function, negative antiphospholipid screen (cardiolipin Ab, anti-beta-2 glycoprotein and DRVVT), negative thrombophilia screen (including Factor V leiden, Antithrombin III, protein C, protein S) and no evidence of uterine structural abnormalities (identified by ultrasound scan or hysteroscopy). Maternal and paternal karyotypes were only carried out if an unbalanced translocation was identified in karyotyped miscarriage tissue as per Royal College of Obstetrics and Gynaecologists guidelines^46^. There was no difference in the age of women between groups: controls 37 ± 4 years versus RM 35 ± 4 years. 30% and 40% had had previous live births respectively. The miscarriage incidence was 0.2 ± 0.1 (2/10 previously had 1 miscarriage) for controls and 3.9 ± 0.3 for RM. Data are displayed as mean ± S.E.M.

### Immune cell isolation from endometrium or peripheral blood

Endometrial samples were obtained using an Endocell disposable endometrial cell sampler (Wallach Surgical devices, CT, USA) under ultrasound guidance starting from the uterine fundus and moving downwards to the internal cervical os.

Samples were washed in PBS to remove blood contamination and tissue cut into 2 mm^3^ fragments before incubation with 1x Liberase (Roche Life Sciences) and 100 μg/ml Deoxyribonuclease 1 (Sigma Aldrich) for 45 minutes at 37 °C with shaking. Digested tissue was resuspended in 10 ml ‘R10’ growth media [Iscove’s Modified Dulbecco’s Medium (Sigma Aldrich), 10% Fetal Calf Serum (FCS) (Gibco), 2% Human Serum (Sera Laboratories International), 1% penicillin-streptomycin (50 IU/ml and 50 μg/ml), 1% glutamine] and filtered through consecutive 70 μm and 40 μm mesh cell strainers (BD Falcon) then cells were washed by centrifugation at 600 × g for 5 minutes.

10 ml peripheral blood was collected into sodium heparin anti-coagulant (10 U/ml) and peripheral blood mononuclear cells (PBMC) isolated using lymphoprep (Axis Shield Diagnostics). PBMC were washed twice in PBS/1% FCS with centrifugation at 800 × g and 200 × g for 10 minutes, then used immediately for flow cytometry.

### Flow cytometry phenotyping

All flow cytometry reagents were from Biolegend unless otherwise stated. Data was acquired using a LSR-II flow cytometer (BD Biosciences) and analysed using FACS DIVA software (BD Biosciences); Fluorescence Minus One (FMO) controls established gating strategies. Figures were generated using FlowJo software (Tree Star Inc.). Immune cells were further purified by separation by density gradient centrifugation over lymphoprep (Axis Shield Diagnostics) then cells were washed twice with PBS/2% FCS and consecutive centrifugation of 800 × g and 200 × g for 10 minutes.

For flow cytometry, cells were incubated with antibodies for 20 minutes at 4 °C, washed ×2 in PBS/2% FCS and fixed in Fluorofix prior to data acquisition. Antibodies used were CD4-FITC(OKT4), CD8a-PE(HIT8a), CD3-PeCy5(UCHT1), CD56-PeCy7(BD Biosciences; B159), CD16-APCCy7(BD Biosciences; 3G8), CD45-APC(HI30), CD103-FITC(BER-ACT8), CD69-PE(FN90), CD8a-PeCy7(RPA-T8), CD45RO-APC-Cy7(UCHL1), CD127-AlexaFluor647(A019D5), PD-1(CD279)-APC(EH12.2H7).

### T cell stimulation and intracellular cytokine staining

For analysis of cytokines produced by intracellular staining flow cytometry, isolated endometrial cells were incubated with 10^6^ CD3/CD28 activator Dynabeads human T-activators CD3/CD28 (Invitrogen) in R10 growth media supplemented with IL-2 (1000 u/ml; Chiron, California, USA) for 5 hours with Brefeldin A addition after 1 hour, then cells were fixed, permeabilised for intracellular staining (BioLegend) and incubated with antibody human IFNγ-FITC (4S.B3) or isotype control mouse IgG1-FITC (ICFC).

### Statistics

All analyses were performed using PRISM software. Differences in cell phenotype between RM and controls were determined using an unpaired Student’s *t*-test. Comparisons of groups of cells within patient groups were analysed with a paired Student’s *t*-test. Normal distribution of data was confirmed using a D’Agostino-Pearson omnibus normality test.

## Additional Information

**How to cite this article**: Southcombe, J. H. *et al*. An altered endometrial CD8 tissue resident memory T cell population in recurrent miscarriage. *Sci. Rep.* **7**, 41335; doi: 10.1038/srep41335 (2017).

**Publisher's note:** Springer Nature remains neutral with regard to jurisdictional claims in published maps and institutional affiliations.

## Supplementary Material

Supplementary Information
