# Supplementary material for: An altered endometrial CD8 tissue resident memory T cell population in recurrent miscarriage
Source: Sci Rep. 2017 Jan 23;7:41335. doi: 10.1038/srep41335 (PMC5256279; doi:10.1038/srep41335)
Supplement: Supplementary Information [file srep41335-s1.pdf]

**An altered endometrial CD8 Tissue Resident memory T cell population in recurrent miscarriage**

J.H.Southcombe, G.Mounce, K.McGee, A.Elghajiji, J.Brosens, S.Quenby, T.Child, I.Granne.

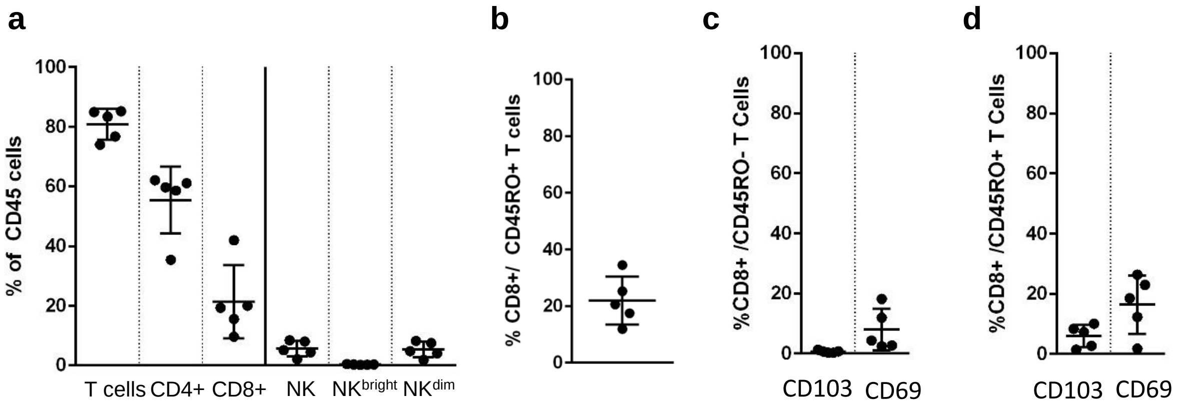


**Supplementary figure 1**

Phenotypic analysis of peripheral blood CD8-T cells (n=5) by flow cytometry. PBMC were isolated and stained with antibodies CD45-AlexaFluor647, CD3-PeCy5, CD4-FITC, CD8-PE, CD56-PeCy7, CD16-APCCy7 and proportions of T and NK cells assessed (a). Alternatively, PBMC were stained with antibodies CD3-PeCy5, CD8-PECy7, CD45RO-APCCy7, CD103-FITC, CD69-PE and the proportion of CD3+/CD8+ cells expressing CD45RO (b), and CD103 or CD69 expression on CD3+CD8+CD45RO- (c) or CD3+CD8+CD45RO+ (d) cells are displayed.
